# Multiple sclerosis and the risk of dementia: a real-world, nationwide cohort study

**DOI:** 10.3389/fneur.2025.1687661

**Published:** 2026-01-12

**Authors:** Ting-An Chen, Sung-Tao Li, Wu-Chien Chien, Chi-Hsiang Chung, Fang-Jung Wan, Ta-Chuan Yeh, Yi-Wei Yeh, Nian-Sheng Tzeng

**Affiliations:** 1 Department of Psychiatry, Tri-Service General Hospital and School of Medicine, College of Medicine, National Defense Medical University, Taipei, Taiwan; 2 Department of Psychiatry, Armed Forces Hualien General Hospital, Hualien, Taiwan; 3 Department of Medical Research, Tri-Service General Hospital and National Defense Medical University, Taipei, Taiwan; 4 School of Public Health, College of Public Health, National Defense Medical University, Taipei, Taiwan; 5 Graduate Institute of Life Sciences, College of Biomedical Sciences, National Defense Medical University, Taipei, Taiwan; 6 Student Counseling Center, National Defense Medical University, Taipei, Taiwan

**Keywords:** multiple sclerosis, dementia, disease-modifying drugs, nationwide cohort study, National Health Insurance Research Database

## Abstract

**Background:**

Multiple sclerosis (MS) is a chronic autoimmune and neurodegenerative disease that often causes cognitive impairment. This study aimed to investigate the association between MS and the risk of dementia in a large, nationwide Taiwanese cohort and to examine the potential impact of Disease-modifying drugs (DMDs) on the dementia incidences.

**Methods:**

We conducted a population-based cohort study using Taiwan’s National Health Insurance Research Database (NHIRD) between 2000 and 2015. Adults aged ≥20 years with a diagnosis of MS (*n* = 10,525) were matched 1:3 by age, sex, index date, and healthcare utilization to non-MS controls (*n* = 31,575). Dementia diagnoses were confirmed ≥1 year after the MS diagnosis. The Fine and Gray competing risks model was applied so as to estimate the sub-distribution hazard ratios (SHRs) for dementia, adjusting for the demographic, socioeconomic, and clinical covariates. Subgroup, sensitivity, and DMD-use analyses were performed.

**Results:**

During the follow-up, the cumulative incidence of dementia was higher in the MS cohort (739.97 per 100,000 person-years) than in the controls (343.95 per 100,000 person-years; log-rank *p* < 0.001). MS was associated with an almost five-fold increased risk of dementia (adjusted SHR = 4.919, 95% CI: 4.329–5.743, p < 0.001). An elevated risk of dementia persisted after excluding the cases diagnosed within the first year and first 5 years. Vascular and autoimmune comorbidities further increased the risk of dementia. The usage of the DMDs, including interferon-β-1a, interferon-β-1b, natalizumab, and teriflunomide was associated with the reduced risk of other degenerative dementias, and the teriflunomide was also linked to a lower Alzheimer’s risk of disease.

**Conclusion:**

MS is an independent and potent risk factor for dementia in the Taiwanese population, from the NHIRD records. The findings underscore the importance of the early cognitive monitoring, the comprehensive comorbidity management, and the optimal pharmacologic intervention. The DMD usage may be associated with a reduced risk of dementia, thereby warranting further prospective investigation.

## Introduction

Multiple sclerosis (MS) is a chronic autoimmune and neurodegenerative disease characterized by the inflammatory demyelination of the central nervous system (CNS), leading to the progressive neurological dysfunction (1, 2). The pathophysiology of MS involves a complex immune dysregulation, including the dendritic cell activation, pro-inflammatory T lymphocyte differentiation, and macrophage and microglial activation, ultimately leading to the myelin destruction and axonal damage (3). The disease is primarily classified into relapsing–remitting (70–80% of cases), primary progressive (10–15%), and secondary progressive (15–20%), with approximately 80–90% of relapsing–remitting patients eventually transitioning to secondary progressive disease within 20–25 years (4).

.While MS is primarily associated with motor and sensory impairments, accumulating evidence suggests that the cognitive dysfunction is a frequent and disabling symptom, affecting approximately 40–65% of MS patients (5). The cognitive deficits in the MS predominantly involve the processing speed, working memory, executive function, and the visuospatial abilities (6). Recent systematic reviews and meta-analyses have demonstrated that the pooled prevalence of the cognitive impairment in the MS patients was 41%, with more pronounced cognitive deficits observed in progressive forms of MS (7). As the most common non-traumatic neurological disabling condition affecting young adults, MS has profound implications for the patients’ quality of life and social functioning (8, 9).

Cognitive impairment in the MS patients may arise through multiple mechanisms, including white and gray matter structural damage, neural network disconnection, neuroinflammation, and synaptic dysfunction (10). Cerebrospinal fluid biomarker studies have demonstrated that the neurofilament light chain was closely associated with the cognitive impairment, particularly the slowed information processing speed (11). Additionally, the concepts of the brain reserve and cognitive reserve provide important frameworks for a better understanding of the heterogeneity cognitive function in the MS patients (12).

Recent epidemiological and neuroimaging studies have highlighted the potential risk factors for dementia in MS, including disease duration, lesion burden, gray matter atrophy, and comorbidities such as cardiovascular disease and metabolic syndrome (13, 14). Furthermore, genetic susceptibility, environmental factors, and lifestyle habits such as smoking and physical inactivity have also been implicated in the progression of cognitive impairment in the MS patients (15).

Large population-based cohort studies in Europe and South Korea have demonstrated a 2.2- to 3.8-fold increased risk of all-cause dementia—including Alzheimer’s disease and vascular dementia—in the MS patients as compared to matched controls (16, 17). However, Taiwan lacks studies leveraging the National Health Insurance Research Database (NHIRD) to quantify the risk of dementia in MS. The NHIRD, covering over 99.7% of the Taiwanese population, offers comprehensive claims data ideal for epidemiological investigations.

Given the clinical importance of cognitive decline in MS, a deeper understanding of the potential transition from MS-related cognitive impairment to dementia is essential. This study aims to explore the risk of dementia in MS patients, identify key predictors, and elucidate possible pathophysiological mechanisms. By identifying the MS patients diagnosed between 2001 and 2016 in the NHIRD and comparing their incidence and hazard ratio (HR) of dementia with matched non-MS controls, we hope to improve the screening, prevention, and management strategies for the cognitive impairment in MS.

## Methods

### Data sources

This nationwide, population-based cohort study utilized the inpatient care records and registration files from Taiwan’s NHIRD, which covers over 99% of the 23 million population since its implementation in 1995. The National Health Insurance (NHI) Administration conducts routine random audits of medical claims so as to ensure diagnostic accuracy. The NHI program and the validity of several NHIRD diagnoses have been documented in previous studies (18, 19).

### Study population

This study employed a cohort design. Using the NHIRD, we selected adult patients aged >20 years diagnosed with dementia between 2000 and 2015. Dementia diagnoses were confirmed by either ≥3 outpatient visits for dementia in consecutive years or ≥1 dementia-related hospitalization during the study period, and all were made by board-certified psychiatrists or neurologists in Taiwan. The date of the dementia diagnosis was required to be at least 1 year after the diagnosis of the MS. This temporal lag was selected to minimize reverse causation bias, allowing sufficient time for MS-related pathophysiological processes to manifest cognitive effects while accounting for MS’s typical diagnostic timeline in claims data. Patients with MS were identified through Taiwan’s catastrophic illness card system, a government-sponsored program that provides financial relief for patients with catastrophic or severe diseases. Catastrophic illness cards require verification by specialists and undergo rigorous review by the National Health Insurance Bureau. MS patients were confirmed through ≥1 hospitalization for MS within the study period or documented MS diagnosis by board-certified neurologists. All the diagnoses of dementia based on the International Classification of Diseases, 9th Revision, Clinical Modification (ICD-9-CM) codes between 2000 and 2015 (shown in Supplementary Table S1). The patients with previous diagnosis of dementia and chronic inflammatory demyelinating polyneuropathy, critical illness polyneuropathy, critical illness myopathy, polyneuropathy because of other diseases such as porphyria and diphtheria, acute poliomyelitis, myasthenia gravis, other myasthenic syndrome, acute transverse myelitis, and poisoning by drugs and biologic substances were excluded (ICD-9-CM codes shown in Supplementary Table S1). Similarly, matched non-MS controls were required to meet the same exclusion criteria as MS patients, including no prior diagnosis of dementia or other chronic neurological conditions, ensuring baseline comparability between the two cohorts.

The index date was defined as MS diagnosis date for MS patients and the first insurance claim within the same calendar year for non-MS controls, ensuring temporal alignment. All baseline characteristics and ages refer to the index date. For each patient with MS included in the study, three matched controls were selected from the NHIRD at a 1:3 ratio, matched by sex, age at index date, index date (same calendar year), and number of medical follow-ups (*N* = 31,575). All insurance claims were reviewed by the medical reimbursement specialists, and peer reviews were conducted in accordance with the standardized clinical diagnostic criteria.

### Covariates

Covariates included sex, age group (20–49, 50–64, and ≥65 years), marital status, education level (<12 years or ≥12 years), insurance premium in New Taiwan Dollars (NT$) (<18,000; 18,000–34,999; or ≥35,000), annual number of medical visits, geographical area of residence (northern, central, southern, eastern Taiwan, or outlying islands), urbanization level of residence (levels 1–4), and level of care. Urbanization level of residence was categorized into 4 levels based on population size and development indicators (Level 1 = highest; Level 4 = lowest), as previously described (20).

Data on the usage of Disease-modifying Drugs (DMDs), including Fingolimod, Glatiramer acetate, Interferon-β-1a, Interferon-β-1b, Natalizumab, and Teriflunomide, were retrieved from the Longitudinal Health Insurance Database (LHID), a sub-database of the NHIRD. DMD exposure was classified as a binary variable (ever-use vs. never-use during follow-up), reflecting medication dispensing records in claims data. However, this approach has inherent limitations characteristic of pharmacoepidemiology studies using administrative databases: the binary classification does not account for treatment duration, cumulative exposure, medication adherence, or switching patterns among DMDs.

### Comorbidity

Comorbidities included cerebrovascular disease, hemiplegia or paraplegia, rheumatologic disease, diabetes, hypertension, hyperlipidemia, coronary artery disease, systemic lupus erythematosus, and rheumatoid arthritis (ICD-9-CM codes as shown in Supplementary Table S1). These comorbidities were selected based on the definitions used in previous studies employing the health database analyses (21, 22).

### Statistical analysis

A Fine and Gray competing risks model was employed to estimate crude and adjusted sub-distribution hazard ratios (SHRs) with corresponding 95% confidence intervals (CIs) for dementia risk. This model was selected because death represents a competing event that precludes dementia diagnosis; individuals who died during the 15-year follow-up period could not develop dementia, and thus standard Cox regression would overestimate the true dementia incidence. By accounting for both the probability of developing dementia and the competing risk of death, sub-distribution hazard ratios provide a more accurate estimate of true dementia incidence in the MS and non-MS cohorts. The primary study endpoint was dementia diagnosis. Stratified analyses by dementia subtype (Alzheimer’s disease, vascular dementia, and other degenerative dementias) were performed to examine whether MS associations differed across dementia phenotypes. The Kaplan–Meier method and log-rank test were employed to assess the differences in the dementia incidences between the participants with and without MS. DMT exposure was classified as a binary variable based on medication dispensing records: ‘ever-use’ (any DMT prescription during follow-up) versus ‘never-use’, representing cumulative exposure status rather than time-dependent modeling. This classification approach is consistent with claims-based pharmacoepidemiological studies. Additionally, the adjusted SHRs were calculated after controlling for covariates, including sex, age group, education level, monthly insured premiums, urbanization level, geographic region, level of care, and comorbidities. Notably, the level of care (medical center, regional hospital, or local hospital) was included as a covariate to account for potential detection bias, as medical centers typically have greater access to advanced neuroimaging, neuropsychological assessment tools, and specialist expertise, which may result in higher rates of dementia diagnosis compared to regional or local hospitals. This adjustment helps mitigate the confounding effect of differential diagnostic intensity across healthcare settings. A *p*-value of <0.05 was considered statistically significant. All data analyses were conducted using the SPSS version 22.0.

## Results

### Enrolled samples

**Figure 1:**
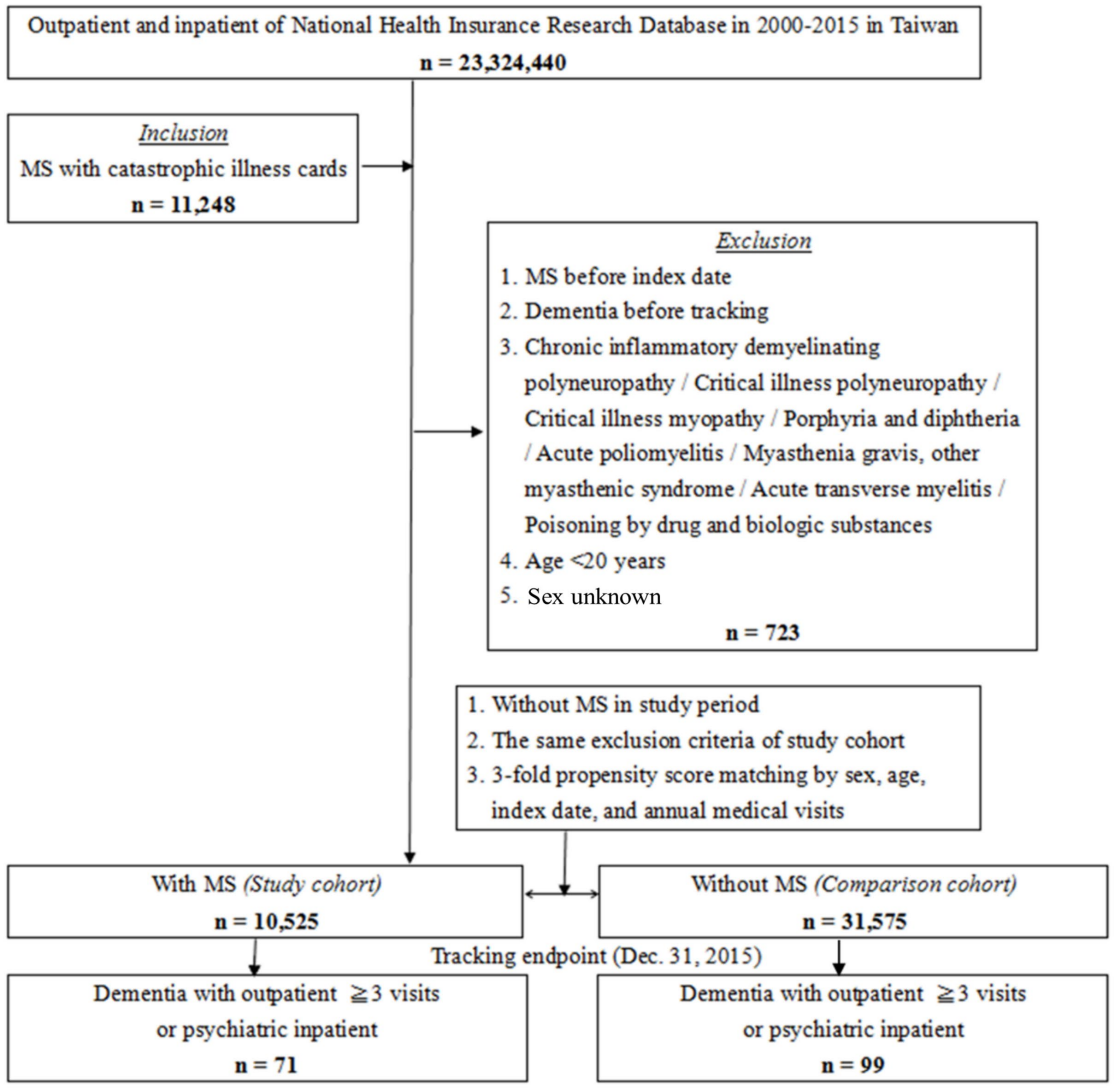
The flowchart of study of multiple sclerosis (MS) sample selection from National Health Insurance Research Database in Taiwan. Both MS patients (*n* = 10,525) and matched non-MS controls (*n* = 31,575) were required to meet identical exclusion criteria, including no prior diagnosis of dementia or chronic neurological conditions detailed in the methods section, ensuring baseline comparability between the two cohorts.

Figure 1 is a flowchart of the patient enrollment procedure. From the NHIRD, we identified 10,525 patients who received a MS diagnosis during our defined study period (2000–2015); these patients were matched 1:3 with patients without MS (*N* = 31,575) according to age, sex, number of visits to medical facilities, and comorbidities.

### Sample characteristics

A total of 10,525 patients were diagnosed with MS during the study period, among whom 71 individuals developed dementia during the follow-up period.

Table 1 shows the sex, age, marital status and comorbidities of the patients with MS. The mean age of the cohort was approximately 41 years, and 80.32% of the participants were female. When compared with the controls, the patients with MS tended to have higher rates of cerebrovascular disease, hemiplegia or paraplegia, rheumatologic disease, hypertension, hyperlipidemia, systemic lupus erythematosus and rheumatoid arthritis.

**Table 1:** Baseline characteristics of participants in the study.

Variables	MS patients (*n* = 10,525)	Control (*n* = 31,575)	*p*-value
Female	8,454 (80.32%)	25,362 (80.32%)	0.999
Age, year	41.19 ± 13.18	40.92 ± 14.68	0.094
Age group			0.999
20–49	7,779 (73.91%)	23,337 (73.91%)	
50–64	2,213 (21.03%)	6,639 (21.03%)	
≧65	533 (5.06%)	1,599 (5.06%)	
Marital status			0.626
Single	8,015 (76.15%)	23,971 (75.92%)	
Married	2,510 (23.85%)	7,604 (24.08%)	
Education (years)			0.481
<12	4,935 (46.89%)	14,930 (47.28%)	
≧12	5,590 (53.11%)	16,645 (52.72%)	
Insurance premium (NT$)			0.957
<18,000	8,500 (80.76%)	25,461 (80.64%)	
18,000-34,999	1,376 (13.07%)	4,147 (13.13%)	
≧35,000	649 (6.17%)	1,967 (6.23%)	
Comorbidities			
Cerebrovascular disease	271 (2.57%)	698 (2.21%)	0.031
Hemiplegia or paraplegia	406 (3.86%)	118 (0.37%)	<0.001
Rheumatologic disease	230 (2.19%)	235 (0.74%)	<0.001
Diabetes	569 (5.41%)	1,633 (5.17%)	0.350
Hypertension	739 (7.02%)	1,865 (5.91%)	<0.001
Hyperlipidemia	422 (4.01%)	134 (0.42%)	<0.001
Coronary artery disease	276 (2.62%)	780 (2.47%)	0.388
Systemic lupus erythematosus	152 (1.44%)	89 (0.28%)	<0.001
Rheumatoid arthritis	43 (0.41%)	77 (0.24%)	<0.001
Annual medical visits, time	9.85 ± 10.27	10.03 ± 11.28	0.775
Location			<0.001
Northern Taiwan	5,418 (51.48%)	13,469 (42.66%)	
Middle Taiwan	2,038 (19.36%)	8,328 (26.38%)	
Southern Taiwan	2,531 (24.05%)	8,267 (26.18%)	
Eastern Taiwan	538 (5.11%)	1,365 (4.32%)	
Outlets islands	0 (0.00%)	146 (0.46%)	
Urbanization level			<0.001
1 (The highest)	5,352 (50.85%)	12,163 (38.52%)	
2	4,421 (42.00%)	12,945 (41.00%)	
3	267 (2.54%)	2,577 (8.16%)	
4 (The lowest)	485 (4.61%)	3,890 (12.32%)	
Level of care			<0.001
Medical center	7,762 (73.75%)	10,012 (31.71%)	
Regional hospital	2,364 (22.46%)	11,177 (35.40%)	
Local hospital	399 (3.79%)	10,386 (32.89%)	

*p*-value: Chi-square/Fisher’s exact test on category variables and t-test on continuous variables. MS: multiple sclerosis; NT$: New Taiwan Dollars; AIDS/HIV: acquired immunodeficiency syndrome/human immunodeficiency virus infection. All values represent status at the index date (MS diagnosis date for MS patients; first claim within same calendar year for non-MS controls). Follow-up extended until December 31, 2015. Annual medical visits = total outpatient and inpatient encounters in the 12 months prior to index date.

Patients with MS also tended to be living in the northern and southern areas of Taiwan and residing more in the regions of urbanization levels 1 and 2. There were no differences in the distribution of sex, age, marital status, education, and insurance premium between these two groups.

### Kaplan–Meier curves for the cumulative incidence of dementia in patients with MS

**Figure 2:**
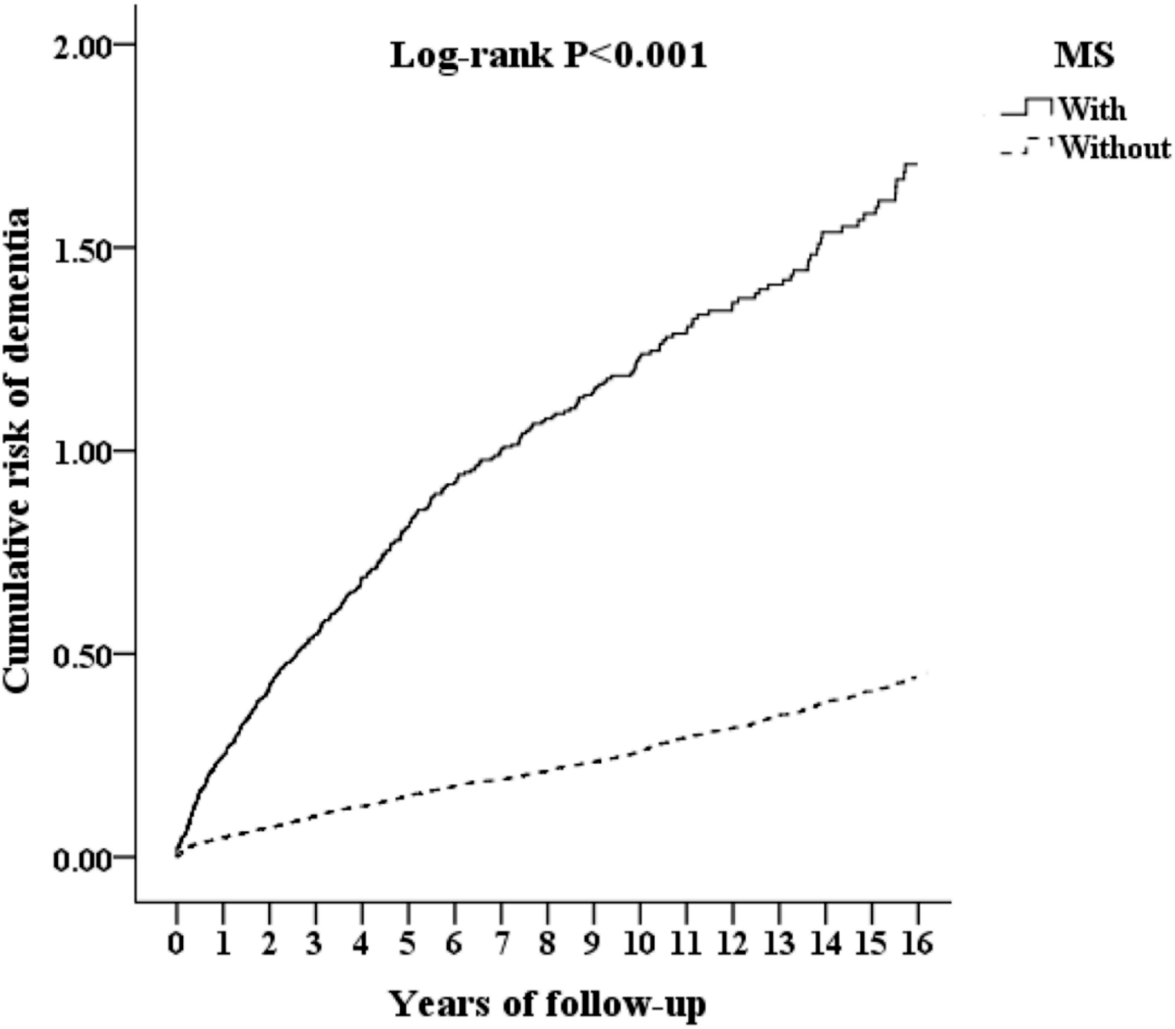
Kaplan–Meier for cumulative risk of dementia aged 20 and over stratified by multiple sclerosis (MS) with log-rank test.

**Figure 3:**
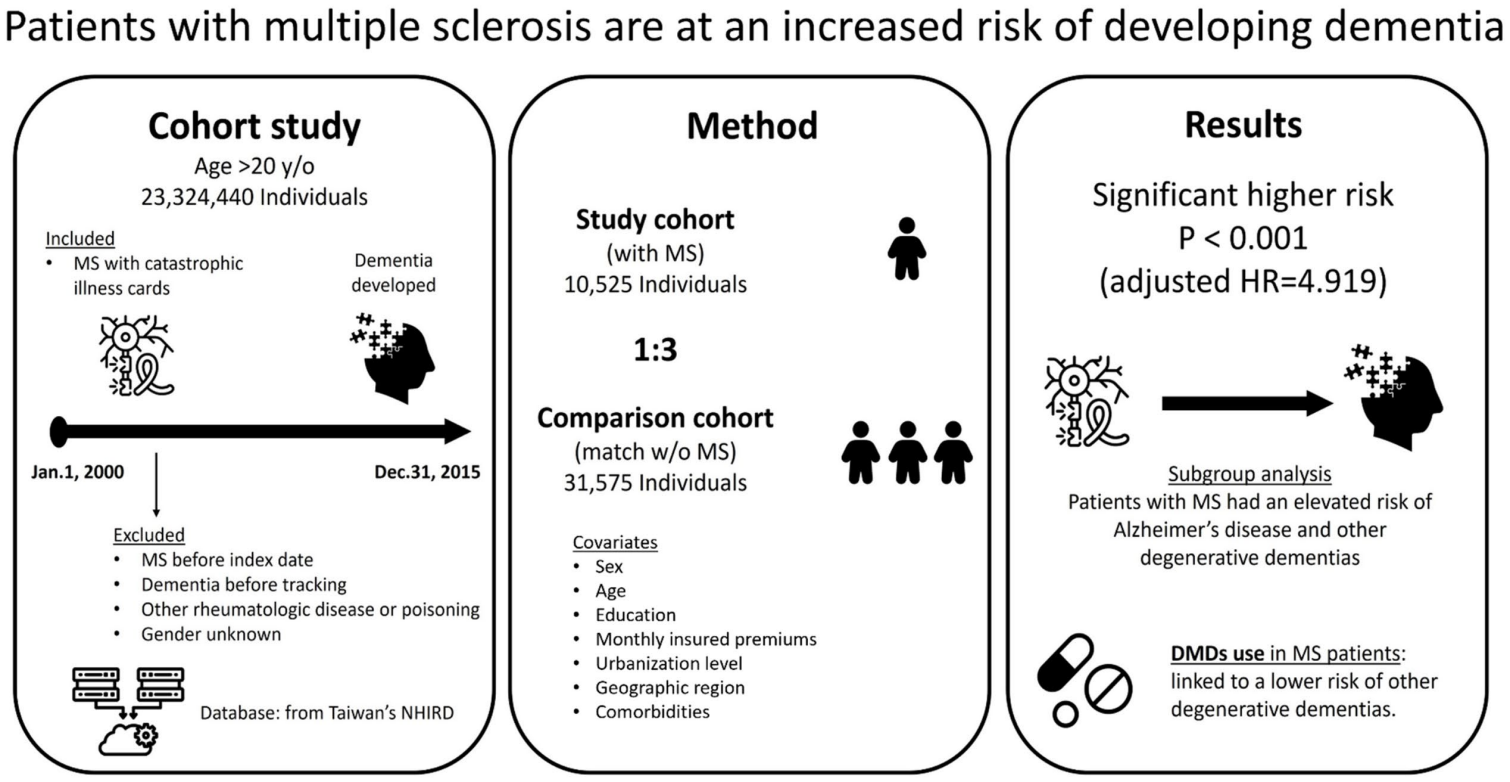
The graphic abstract of study design and results from the National Health Insurance Research Database in Taiwan. MS, multiple sclerosis; HR, hazard ratio; DMD, disease-modifying drugs. All icons are from the Noun Project.

The cumulative incidence of dementia was 739.97 per 100,000 person-years in the MS cohort and 343.95 per 100,000 person-years in the comparison cohort (Table 2). The Kaplan–Meier analysis demonstrated a significantly higher cumulative risk of dementia among the individuals with MS as compared to those without MS (log-rank test, *p* < 0.001; Figure 2). Figure 3 shows the graphic abstract of the study design and the results from the NHIRD in Taiwan for the MS and comparison groups.

**Table 2:** Factors of dementia by using Fine & Gray’s competing risk model.

Variables	Competing risk in the model							
	Crude SHR	95% CI	95% CI	*p*	Adjusted SHR	95% CI	95% CI	*p*
Multiple sclerosis (reference: without)	4.298	3.813	4.977	<0.001	4.919	4.329	5.743	<0.001
Dementia with Multiple sclerosis (71/10,525; 739.97 per 10^5^ person-years)								
Dementia without Multiple sclerosis (99/31,575; 343.95 per 10^5^ person-years)								
Male (reference: female)	0.744	0.655	0.872	<0.001	0.853	0.747	0.973	0.022
Peripheral vascular disease (reference: without)	8.178	2.026	33.913	<0.001	5.227	1.274	22.030	<0.001
Cerebrovascular disease (reference: without)	1.609	1.302	2.044	<0.001	1.434	1.132	1.865	<0.001
Chronic pulmonary disease (reference: without)	1.763	1.395	2.290	<0.001	1.870	1.464	2.456	<0.001
Peptic ulcer disease (reference: without)	1.734	1.407	2.195	<0.001	1.564	1.259	1.996	<0.001
Diabetes mellitus (reference: without)	1.648	1.415	1.971	<0.001	1.517	1.286	1.838	<0.001
Renal disease (reference: without)	3.323	2.444	4.641	<0.001	3.426	2.494	4.837	<0.001
Liver disease (reference: without)	1.944	1.615	2.406	<0.001	2.085	1.716	2.603	<0.001
Hyperlipidemia (reference: without)	2.202	1.798	2.772	<0.001	1.708	1.367	2.193	<0.001
Coronary artery disease (reference: without)	1.899	1.603	2.310	<0.001	1.625	1.351	2.007	<0.001
Systemic lupus erythematosus (reference: without)	2.083	1.301	3.427	<0.001	1.773	1.100	2.938	<0.001
Rheumatoid arthritis (reference: without)	2.499	1.229	5.220	<0.001	2.902	1.420	6.096	<0.001
Deficiency anemias (reference: without)	1.672	1.209	2.374	<0.001	2.330	1.675	3.332	<0.001
Fluid and electrolyte disorders (reference: without)	1.841	1.492	2.334	<0.001	1.502	1.204	1.924	<0.001
Urbanization Level 1 (reference: without)	0.764	0.792	1.005	0.055	0.812	0.672	1.010	0.059
Medical center (reference: without)	1.175	1.019	1.390	0.001	1.826	1.703	2.019	<0.001

SHR, Subdistribution Hazard ratio; Adjusted for demographic variables (sex, age, education, urbanization, region), socioeconomic factors (insurance premium, level of care), and comorbidities (detailed in Methods and Supplementary Table S1). CI, confidence interval.

### Hazard ratios analysis of dementia in the patients with MS

In our applied Fine and Gray competing risk model (Table 2), individuals with MS had a five-fold increased risk of dementia as compared to those without MS (crude SHR 4.298, 95% CI: 3.813–4.977, *p* < 0.001). After we adjusted for the covariates, including sex, age group, education level, monthly insured premiums, urbanization level, geographic region, and comorbidities, the adjusted SHR for MS individuals was 4.919 (95% CI: 4.329–5.743, *p* < 0.001).

In addition, we observed that the patients with several comorbid conditions were associated with an increased risk of dementia. Notably, the individuals with peripheral vascular disease exhibited the highest risk, with an adjusted SHR of 5.227 (95% CI: 1.274–22.030, p < 0.001). Other comorbidities associated with a relatively elevated risk of dementia included vascular conditions (e.g., cerebrovascular disease, coronary artery disease, hyperlipidemia), metabolic and gastrointestinal disorders (e.g., diabetes mellitus, liver disease, peptic ulcer disease), renal and electrolyte imbalances (e.g., renal disease, fluid and electrolyte disorders), chronic pulmonary disease, deficiency anemias, and autoimmune or inflammatory conditions (e.g., systemic lupus erythematosus, rheumatoid arthritis). Furthermore, receiving care from a medical center was associated with a higher risk of dementia. In contrast, males were associated with a reduced risk of dementia.

### Subgroup and sensitivity analysis in the risk of dementia in MS

Patients with MS exhibited an elevated risk of multiple dementia subtypes, including Alzheimer’s disease and other degenerative dementias, as compared with the controls. This increased the overall risk that remained significant even after excluding the dementia cases diagnosed within the first year and the first 5 years of follow-up (Table 3).

**Table 3:** Factors of dementia subgroup and sensitivity analysis by using Fine & Gray’s competing risk model.

Sensitivity analysis	MS (With vs. Without)	(N in the MS patients)	No competing risk in the model				Competing risk in the model			
	Dementia subgroup		Adjusted HR	95% CI	95% CI	*p*	Adjusted SHR	95% CI	95% CI	*p*
Overall	Overall	71	4.298	3.813	4.977	<0.001	4.919	4.329	5.743	<0.001
	Alzheimer dementia	8	5.861	4.121	6.985	<0.001	6.003	4.462	7.011	<0.001
	Vascular dementia	12	2.095	0.986	2.978	0.067	2.249	0.991	3.270	0.059
	Other degenerative dementia	51	3.121	2.860	4.765	<0.001	3.258	2.897	4.796	<0.001
First year diagnoses excluded	Overall	66	3.252	2.849	3.781	<0.001	3.288	2.870	3.864	<0.001
	Alzheimer dementia	7	4.201	3.064	5.201	<0.001	4.225	3.075	5.218	<0.001
	Vascular dementia	11	1.531	0.845	2.206	0.165	1.546	0.859	2.244	0.142
	Other degenerative dementia	48	2.864	1.975	3.489	<0.001	2.870	1.982	3.496	<0.001
First 5 years diagnoses excluded	Overall	49	2.723	2.386	3.166	<0.001	2.869	2.397	3.257	<0.001
	Alzheimer dementia	5	3.121	2.567	4.298	<0.001	3.134	2.578	4.301	<0.001
	Vascular dementia	8	1.089	0.718	2.179	0.288	1.093	0.724	2.184	0.275
	Other degenerative dementia	36	2.115	1.576	3.010	<0.001	2.128	1.580	3.022	<0.001

HR, Hazard ratio; SHR, Subdistribution Hazard ratio; CI Confidence interval (lower and upper 95% confidence limits); Adjusted for demographic variables (sex, age, education, urbanization, region), socioeconomic factors (insurance premium, level of care), and comorbidities (detailed in Methods and Supplementary Table S1). CI, confidence interval.

### DMDs and the risk of dementia in patients with MS

Table 4 presents the associations between the usage of the DMDs and the risk of dementia among patients with MS. The dementia subgroup was classified into Alzheimer’s disease, vascular dementia, and other degenerative dementias. Among the DMDs examined, the usage of interferon-β-1a (95% CI: 0.316–0.966, *p* = 0.015), interferon-β-1b (95% CI: 0.317–0.970, *p* = 0.011), natalizumab (95% CI: 0.530–0.992, *p* = 0.046), and teriflunomide (95% CI: 0.358–0.936, *p* < 0.001) were significantly associated with a reduced risk of other degenerative dementias. Furthermore, teriflunomide usage was also associated with a decreased risk of Alzheimer’s disease (95% CI: 0.417–0.982, *p* = 0.036). Overall, the usage of DMDs among patients with MS was linked to a lower risk of developing other degenerative dementias (95% CI: 0.185–0.997, *p* = 0.043).

**Table 4:** Factors of subgroup in different disease-modifying drugs among multiple sclerosis patients by using Cox regression and Fine & Gray’s competing risk model.

Drugs (Reference: Without)	Model	No competing risk in the model				Competing risk in the model			
	Dementia subgroup	Adjusted HR	95% CI	95% CI	*p*	Adjusted SHR	95% CI	95% CI	*p*
Overall	Overall	0.675	0.428	1.100	0.406	0.692	0.439	1.128	0.416
	Alzheimer dementia	0.580	0.225	1.297	0.135	0.594	0.230	1.329	0.138
	Vascular dementia	0.741	0.518	1.466	0.206	0.759	0.531	1.502	0.211
	Other degenerative dementia	0.508	0.180	0.986	0.038	0.520	0.185	0.997	0.043
Fingolimod	Overall	0.751	0.575	1.204	0.360	0.770	0.589	1.233	0.369
	Alzheimer dementia	0.698	0.247	1.513	0.271	0.716	0.253	1.550	0.277
	Vascular dementia	0.778	0.581	1.991	0.498	0.798	0.596	2.040	0.510
	Other degenerative dementia	0.596	0.238	1.050	0.090	0.611	0.244	1.076	0.092
Glatiramer acetate	Overall	0.638	0.463	1.200	0.140	0.653	0.474	1.229	0.143
	Alzheimer dementia	0.525	0.161	1.332	0.153	0.538	0.165	1.365	0.157
	Vascular dementia	0.795	0.593	1.729	0.285	0.814	0.608	1.772	0.292
	Other degenerative dementia	0.476	0.172	0.992	0.039	0.488	0.176	1.016	0.054
Interferon-β-1a	Overall	0.692	0.415	1.352	0.262	0.709	0.425	1.386	0.268
	Alzheimer dementia	0.619	0.357	1.001	0.051	0.635	0.366	1.026	0.052
	Vascular dementia	0.712	0.471	2.033	0.712	0.729	0.482	2.083	0.729
	Other degenerative dementia	0.570	0.309	0.943	0.003	0.584	0.316	0.966	0.015
Interferon-β-1b	Overall	0.796	0.427	1.286	0.226	0.815	0.438	1.317	0.232
	Alzheimer dementia	0.540	0.377	1.009	0.059	0.554	0.386	1.034	0.060
	Vascular dementia	0.874	0.569	1.317	0.807	0.895	0.583	1.350	0.827
	Other degenerative dementia	0.489	0.310	0.947	0.001	0.501	0.317	0.970	0.011
Natalizumab	Overall	0.724	0.560	1.091	0.430	0.742	0.574	1.118	0.440
	Alzheimer dementia	0.814	0.606	1.054	0.090	0.834	0.620	1.080	0.092
	Vascular dementia	0.896	0.680	1.765	0.391	0.918	0.697	1.808	0.401
	Other degenerative dementia	0.712	0.517	0.985	0.038	0.729	0.530	0.992	0.046
Teriflunomide	Overall	0.836	0.403	2.964	0.490	0.857	0.413	3.037	0.502
	Alzheimer dementia	0.673	0.407	0.958	0.011	0.690	0.417	0.982	0.036
	Vascular dementia	0.988	0.511	3.267	0.712	1.013	0.524	3.348	0.729
	Other degenerative dementia	0.509	0.349	0.913	<0.001	0.521	0.358	0.936	<0.001

HR, Hazard ratio; SHR, Subdistribution Hazard ratio; Adjusted for demographic variables (sex, age, education, urbanization, region), socioeconomic factors (insurance premium, level of care), and comorbidities (detailed in Methods and Supplementary Table S1). CI, confidence interval.

### Supplementary note on DMD analysis

Our DMD-analysis examined binary exposure (exposed vs. non-exposed) during the entire follow-up period. While we observed that DMD usage was associated with reduced dementia risk, particularly for other degenerative dementias (adjusted SHR = 0.520, 95% CI 0.185–0.997, p = 0.043) and specifically for teriflunomide with Alzheimer’s disease risk reduction (adjusted SHR = 0.690, 95% CI 0.417–0.982, *p* = 0.036), this analysis does not differentiate outcomes based on treatment duration, medication adherence, or DMD-switching patterns. Therefore, the observed associations may reflect a combination of pharmacological effects and healthy-user bias.

## Discussion

### The association between MS and the risk of dementia

By employing a large, nationwide population-based cohort and utilizing the Fine and Gray competing risk model, our study is the first to confirm a significantly increased risk of subsequent dementia in Taiwanese patients with MS. Individuals with MS exhibited an almost five-fold higher risk of developing dementia (adjusted SHR = 4.919, 95% CI: 4.329–5.743, *p* < 0.001) as compared to matched non-MS controls, after adjusting for key confounders such as age, sex, socioeconomic status, urbanization, geographic region, and common comorbidities. These findings are consistent with those of previous large-scale studies conducted in Europe and Asia, all of which have similarly reported a substantially elevated all-cause and subtype-specific dementia risk among MS patients (16, 23).

The cumulative incidence of dementia was 739.97 per 100,000 person-years in the MS cohort—more than double the 343.95 per 100,000 person-years observed in non-MS controls—as demonstrated by a clear divergence in cumulative risk on Kaplan–Meier analysis. The apparent discrepancy between the person-years incidence rate ratio (approximately 2.1) and the crude sub-distribution hazard ratio (SHR = 4.3) warrants explanation. This difference arises from several methodological considerations: First, sub-distribution hazard ratios from the Fine and Gray competing risks model account for the competing event of death, which removes individuals from the dementia risk set. Second, the person-years calculation uses simple event rates and does not account for censoring or competing events. Third, the competing risks framework estimates the probability of dementia conditional on not having died, which can yield larger effect estimates than simple incidence rate ratios. This methodological distinction does not invalidate either approach but reflects the different information each method provides.

Notably, comorbidities such as peripheral vascular disease (adjusted SHR = 5.227, 95% CI: 1.274–22.030, *p* < 0.001), cerebrovascular and coronary artery disease, diabetes, hepatic and renal dysfunction, chronic pulmonary disease, anemia, and other autoimmune or inflammatory disorders were all independently associated with an increased dementia risk in both the MS and non-MS subjects. Our results highlighted the importance of the comprehensive management of comorbidities in the MS patients so as to mitigate the subsequent neurodegenerative risk. Even after excluding the dementia cases diagnosed within the first year and the first 5 years of follow-up, patients with MS continued to exhibit an elevated risk of dementia. This nationwide study provides a comprehensive assessment of the association between MS and dementia, demonstrating a persistently increased risk among the individuals with MS.

In terms of therapy, the usage of the DMDs—including Interferon-β-1a, Interferon-β-1b, Natalizumab, and Teriflunomide—was significantly associated with a decreased risk of other degenerative dementias among the MS patients, and Teriflunomide usage was additionally linked to a lower risk of Alzheimer’s disease. This supports the neuroprotective role of DMDs and the importance of the optimal pharmacological management to potentially slow the cognitive decline and reduce the dementia incidence in MS. This finding might be one of the first evidence supporting that the DMDs could be associated with the reduced risk of MS-related, degenerative dementia. Furthermore, seeking care in medical centers was associated with a higher risk of dementia, possibly reflecting the referral of more severe cases. In contrast, males were a protective factor for dementia among individuals with MS.

Our sensitivity analyses support a genuine temporal relationship between MS and dementia rather than reverse causation. Even after excluding dementia cases diagnosed within the first year (adjusted SHR = 3.288, Table 3) and the first 5 years post-MS diagnosis (adjusted SHR = 2.869), MS remained significantly associated with elevated dementia risk. These findings support that cognitive decline is attributable to MS pathology rather than pre-existing cognitive impairment driving MS diagnostic workup. The one-year minimum interval reflects the typical MS diagnostic timeframe and allows sufficient biological plausibility for MS pathological processes to initiate cognitive impairment. However, residual confounding from occult cognitive impairment at the time of MS diagnosis cannot be entirely excluded, particularly in claims-based databases lacking standardized neuropsychological assessments. The use of 1-year and 5-year lag-time windows aligns with established pharmacoepidemiologic practices in cohort studies examining disease-outcome associations (24, 25). Multiple recent studies have employed similar lag strategies: a Danish study examining opioid use and dementia utilized both 1-year lag and 5-year lag periods to assess reverse causation (26), while a cohort study of proton pump inhibitors and dementia risk applied standard 5-year lag windows with sensitivity analyses at 2-year and no-lag intervals (27). The rationale for selecting 1-year and 5-year lag periods rather than 3-year intervals reflects standard practice in dementia epidemiology, where researchers typically compare shorter lag periods (1–2 years, addressing diagnostic misclassification and immediate confounding) versus longer lag periods (5 + years, accounting for disease latency and biological plausibility) (28). The fact that MS-dementia associations persist across both lag periods strengthens the inference of causality and substantially reduces the likelihood of reverse causation.

An important consideration in interpreting our findings is the disparity in healthcare level between the MS and non-MS cohorts. As shown in Table 1, a significantly higher proportion of MS patients received care at medical centers compared to controls (73.75% vs. 31.71%, *p* < 0.001). Medical centers in Taiwan are equipped with advanced diagnostic resources, including high-resolution neuroimaging (e.g., 3 T MRI), comprehensive neuropsychological testing batteries, and multidisciplinary teams specializing in neurodegenerative disorders, whereas regional and local hospitals have more limited access to such resources. This differential diagnostic intensity may introduce detection bias, wherein the observed elevated dementia risk in MS patients could be partially attributable to more rigorous screening and earlier diagnosis.

However, even after adjusting for the level of care in our multivariable Fine and Gray competing risk model, MS patients demonstrated a persistently elevated dementia risk (adjusted SHR = 4.919, 95% CI 4.329–5.743, *p* < 0.001). This robust association supports the conclusion that MS is an independent risk factor for dementia. Furthermore, the finding that receiving care at medical centers was independently associated with higher dementia risk (adjusted SHR = 1.826, Table 2) reinforces the role of healthcare setting as a proxy for diagnostic intensity. These findings suggest that the MS-dementia association is not solely explained by detection bias. Future studies incorporating standardized cognitive assessments and uniform diagnostic criteria across healthcare settings are needed to further clarify whether this association reflects true biological mechanisms or partially results from diagnostic practice variation.

An interesting observation is the absence of MS cases in Taiwan’s outlying islands region. This likely reflects the pattern of healthcare-seeking behavior in these regions, where patients with suspected neurological conditions typically travel to major medical centers on mainland Taiwan for diagnosis and management. Alternatively, the lower population density in these regions may result in insufficient prevalence of MS to observe cases in the study sample. This geographic pattern does not compromise the internal validity of our findings, as the matching algorithm ensured comparability between MS and non-MS participants within the same geographic categories.

Our study provides strong evidence that MS is an independent and potent risk factor for dementia in the East Asian population. These findings reinforce the need for early intervention, tailored disease management, and further research into the pathophysiological mechanisms and preventive strategies for the cognitive decline in MS.

### Comparison of this study to previous literatures

Our study demonstrates that Taiwanese patients with MS face a significantly increased risk of developing dementia as compared to the general population, with an adjusted SHR of 4.919—higher than previously reported figures from Western or other Asian cohort studies. For example, Cho et al. (16) using the South Korean National Health Insurance database, reported an adjusted HR for all-cause dementia, Alzheimer’s disease, and vascular dementia of 2.34, 2.23, and 3.75, respectively, in MS patients versus matched controls. Large-scale studies from the US and UK similarly found HRs for incident dementia in people with MS ranging from 1.56–2.87, reaffirming the robust and internationally consistent association between MS and dementia (1, 16, 29–31).

Furthermore, our findings that vascular comorbidities, especially peripheral vascular disease, are strong predictors of dementia risk echo those from Britain, South Korea, and US studies, which have shown that even after controlling for lifestyle and comorbidity, MS itself remains an independent dementia risk factor (1, 16, 30).

Distinct from some early perceptions that MS produces cognitive impairment but rarely meets the criteria for major dementia, our study’s longitudinal cohort design confirms a much higher dementia incidence, with both Alzheimer’s disease and vascular dementia being significantly increased—findings strongly paralleling the recent nationwide results from South Korea and the UK (16, 30). Leveraging Taiwan’s nationwide, nearly complete health claims data, the scale and follow-up length of our study also exceeded many published reports, suggesting that the dementia risk in East Asian MS populations may even be higher than in some Western cohorts.

Recent systematic reviews and large-scale data from the US reinforced our findings, showing that MS patients are at persistently increased risk for both early- and late-onset dementia even after adjusting for comorbidities, healthcare access, and demographic factors (1, 29, 30, 32).

### How MS might increase the risk of dementia

MS is a chronic autoimmune and neurodegenerative disorder characterized by inflammatory demyelination and progressive neuronal damage in the CNS. The mechanisms by which MS may increase the risk of dementia involves multiple converging pathological processes.

#### Cortical and gray matter demyelination disrupts higher-order cognitive networks

MS causes extensive subpial and intracortical demyelination that directly impairs limbic and associative cortices, thereby accelerating the dementia phenotypes (33). A 20-year longitudinal MRI study found that early volume loss in regions such as the precuneus, insula, para-hippocampal gyrus, and cingulate independently predicted severe global and domain-specific cognitive impairment two decades later, underscoring cortical atrophy as an initiator of dementia progression in MS (34).

#### Thalamus injury drives information-processing failure

Multi-center real-world data show that a lower baseline thalamic volume and a greater thalamic dysconnectivity predict faster Symbol Digit Modalities Test decline over 3 years, making thalamic injury a pivotal substrate for MS-related dementia (35, 36).

#### Chronic neuroinflammation and synaptopathy

Persistent microglial activation releases cytokines such as IL-1β and TNF-α, creating a neuroinflammatory milieu that drives the synaptic dysfunction and oxidative stress—processes implicated in both MS neurodegeneration and various dementias (37, 38). Aging-related immunosenescence further amplifies this inflammatory cascade, linking MS pathology to age-dependent cognitive decline (16).

#### Axonal damage biomarker—serum neurofilament light chain (Nf-L)

Serum NfL (sNfL) reflects the acute and subacute axonal damage. In secondary-progressive MS, each doubling of baseline sNfL predicted a 0.24-point decline in Full-Scale IQ Z-score over 24 months, outperforming the MRI volumetrics (39). Elevated sNfL is also cross-sectionally and longitudinally associated with a slower processing speed and poorer memory, indicating that ongoing axonal degeneration is a key mediator of dementia risk in MS (40).

#### Vascular comorbidities and small-vessel disease (SVD) synergy

Peripheral vascular disease, hypertension, diabetes, and dyslipidemia are common in MS and accelerate cognitive decline (41). MRI and pathology studies reveal that cerebral SVD can overlap the MS lesions, causing chronic hypoperfusion and hypoxia that potentiate demyelination and neurodegeneration (42, 43). Canonical correlation analyses show that the vascular comorbidities partially mediate the link between MS and lower cognition via macro- and micro-structural brain changes, particularly increasing the risk of vascular dementia (44).

In summary, multifocal demyelination in cortical and deep gray matter, chronic neuroinflammation, progressive axonal damage, and vascular comorbidities synergistically contribute to the increased risk of dementia observed in MS patients.

### Limitations

This cohort study utilizing Taiwan’s NHIRD to investigate the relationship between MS and dementia risk, while benefiting from the national population coverage and long-term follow-up, has several important limitations that warrant careful consideration.

First, diagnostic accuracy represents a primary concern, as the NHIRD primarily relies on the ICD coding from insurance claims to identify diseases, potentially leading to misclassification bias where some MS or dementia cases may be incorrectly categorized due to diagnostic criteria variations, incomplete clinical documentation, or coding errors. Although the catastrophic illness registry undergoes expert review for higher accuracy, routine outpatient diagnoses may still be influenced by variations in diagnostic practices among medical institutions and physicians, particularly regarding the MS subtype classification and dementia subtype identification.

Second, a notable limitation is the significant disproportion in the level of care between MS and non-MS cohorts, with 73.75% of MS patients receiving care at medical centers compared to only 31.71% of controls. Although we adjusted for the level of care in our multivariable models, this adjustment cannot eliminate potential detection bias. Medical centers have substantially greater diagnostic resources and subspecialty expertise, which may result in higher rates of dementia identification among MS patients due to heightened clinical surveillance rather than true biological risk differences. Critically, the inability to access standardized neuropsychological test scores, uniform cognitive screening protocols, or brain MRI volumetric measurements substantially limits our capacity to distinguish genuine biological risk from diagnostic ascertainment bias and residual confounding from unmeasured differences in diagnostic practices across healthcare settings.

Third, the lack of crucial clinical information limits the possibility of in-depth analysis, as the NHIRD cannot provide neuropsychological test scores, MRI imaging changes, disease severity assessments (such as EDSS scores), age of onset, disease duration, and other important clinical parameters, thereby constraining mechanistic analyses between the different MS types, cognitive trajectories, and dementia subtypes.

Fourth, inadequate control of residual confounding factors may affect the result’s credibility; although the study adjusted for multiple demographic and comorbidity variables, important influencing factors such as education level, lifestyle habits (smoking, alcohol consumption, physical activity), social support systems, and family history are not comprehensively recorded in the NHIRD, potentially leading to an insufficient confounding control and affecting causal inference.

Fifth, the binary classification of DMD exposure (exposed vs. non-exposed) substantially simplifies the complexity of real-world pharmacotherapy. The NHIRD captures medication dispensing but cannot assess actual treatment adherence, cumulative drug exposure, or duration of consistent use. Consequently, our analysis cannot differentiate between patients with sustained, adherent DMD therapy versus those with sporadic or poor adherence, both classified as “exposed.” Additionally, the analysis does not account for DMD-switching patterns, which are common in MS management and may have independent effects on cognitive outcomes. Furthermore, dose–response relationships between treatment duration and dementia risk reduction could not be examined. These limitations preclude mechanistic conclusions about whether the observed reduced dementia risk reflects true neuroprotective effects of DMDs or selection bias from healthier, more adherent patients.

Sixth, our dementia subtype classification relied exclusively on ICD-9-CM codes, which have important limitations. These codes primarily reflect clinical documentation rather than neuropathological confirmation. Mixed dementia, involving overlapping pathologies, cannot be reliably distinguished using administrative codes and may be arbitrarily categorized. Moreover, the heterogeneous “other degenerative dementia” category (ICD-9-CM 290.9) includes multiple distinct conditions such as Lewy body and frontotemporal dementia with different etiologies. Consequently, apparent differences in dementia subtype-specific associations with MS should be interpreted with caution, as they may partly reflect coding practices rather than true biological differences.

Seventh, data completeness and follow-up quality issues arise when patients emigrate, die, transfer to non-NHI healthcare systems, or experience difficulties in case identification due to insurance changes during the follow-up period, potentially causing loss-to-follow-up bias and underestimating or overestimating true dementia incidence rates.

Eighth, this study utilized data through December 31, 2015. The follow-up period extended through December 31, 2015, representing the most recent data available at the time of this study from the NHIRD. Due to standard data release protocols and privacy protection regulations, a lag period of approximately 1–2 years is typically required before de-identified claims data becomes available for research use. Consequently, more recent data beyond 2015 were not yet publicly accessible at the time of study design and analysis. Future studies incorporating more recent follow-up periods would provide updated epidemiological insights.

Ninth, our subgroup and DMD-specific analyses presented in Tables 3, 4 involved multiple statistical comparisons without formal correction for multiple testing (e.g., Bonferroni adjustment). Several associations showed borderline statistical significance with 95% confidence intervals approaching or overlapping unity (e.g., Interferon-β-1a adjusted SHR = 0.584, 95% CI 0.316–0.966; Natalizumab adjusted SHR = 0.729, 95% CI 0.530–0.992). These findings should be interpreted cautiously and considered hypothesis-generating rather than definitive evidence. The small number of dementia cases within DMD subgroups limits statistical power. Future larger prospective studies are needed to confirm these preliminary observations and establish the true neuroprotective efficacy of individual DMDs.

Tenth, limitations in external validity must be considered, as Taiwan’s population structure, healthcare system, and disease patterns differ from those in Western countries, requiring caution when extrapolating study results to other populations or healthcare systems, and the inability to access biomarkers, neuroimaging, or genetic data limits direct exploration of pathological mechanisms.

## Conclusion

This study confirmed that the individuals with MS had a nearly five-fold increased risk of developing dementia as compared to those without MS, which should alert physicians to be more attentive to the risk of dementia following MS. Besides, we noted that the risk of some degenerative dementia could be reduced among the MS patients when the DMDs were administered. We recommend the execution of additional studies based on the extensive or national data sets to corroborate the present findings and elucidate the corresponding underlying mechanisms.

## Data Availability

The data are not publicly available due to NHIRD data regulations and privacy protection policies, but can be accessed through the official application process.
