# Supplementary material for: Multiple sclerosis and the risk of dementia: a real-world, nationwide cohort study
Source: Front Neurol. 2026 Jan 12;16:1687661. doi: 10.3389/fneur.2025.1687661 (PMC12832492; doi:10.3389/fneur.2025.1687661)
Supplement: Supplementary file 1 [file Data_Sheet_1.pdf]

Table S1. International Classification of Diseases, 9th Revision, Clinical Modification

	ICD-9-CM
**Study population**	
Multiple sclerosis	340
**Disease excluded:**	
Chronic inflammatory demyelinating polyneuropathy	357.81
Critical illness polyneuropathy	357.82
Critical illness myopathy	359.81
Porphyria and diphtheria	357.4
Acute poliomyelitis	045, 045.1
Myasthenia gravis, other myasthenic syndrome	358.0, 358.01, 358.1, 358.8
Acute transverse myelitis	323
Poisoning by drug and biologic substances	960, 979
**Events**	
dementia	
	Alzheimer dementia (ICD-9-CM codes: 290.0, 290.10, 290.11, 290.12, 290.13, 290.20, 290.21, 290.3, 331.0)<br>Vascular dementia (ICD-9-CM codes: 290.41, 290.42, 290.43)<br>Other degenerative dementia (ICD-9-CM codes: 290.9)
**Comorbidities**	
Cerebrovascular disease	430-438
Hemiplegia or paraplegia	342.9, 344.1

Rheumatologic disease	397.9, 729.0
Diabetes mellitus	250
Hypertension	401-405
Hyperlipidemia	272
Coronary artery disease	414
Systemic lupus erythematosus	710.0
Rheumatoid arthritis	714.0

ICD-9-CM = International Classification of Diseases, 9th Revision, Clinical Modification
